# (*Z*)-3-({[3-Meth­oxy-5-(tri­fluoro­meth­yl)phen­yl]imino}meth­yl)benzene-1,2-diol

**DOI:** 10.1107/S2056989019003220

**Published:** 2019-03-26

**Authors:** Sibel Demir Kanmazalp, Onur Erman Doĝan, Necmi Dege, Erbil Aĝar, Hakan Bulbul, Irina A. Golenya

**Affiliations:** a Gaziantep University, Technical Sciences, 27310, Gaziantep, Turkey; b Ondokuz Mayıs University, Faculty of Arts and Sciences, Department of Chemistry, 55139, Kurupelit, Samsun, Turkey; c Ondokuz Mayıs University, Faculty of Arts and Sciences, Department of Physics, 55139, Kurupelit, Samsun, Turkey; d Taras Shevchenko National University of Kyiv, Department, of Chemistry, 64, Vladimirska Str., Kiev 01601, Ukraine

**Keywords:** crystal structure, Schiff base, O⋯O inter­action, Hirshfeld surface analysis

## Abstract

The title compound, C_15_H_12_F_3_NO_3_, crystallizes with one mol­ecule in the asymmetric unit. The mean planes of the two phenyl rings of the Schiff base moiety, bearing the OH groups and the imine group, respectively, are inclined to each other by 4.91 (1)°. In the crystal, mol­ecules are linked *via* pairs of bifurcated O—H⋯O hydrogen bonds between the phenol OH groups, forming inversion dimers with an *R*_1_^2^(5) ring motif. The structure exhibits also intra­molecular O—H⋯N and C—H⋯F hydrogen-bonding inter­actions. Hirshfeld surfaces analysis and two-dimensional fingerprint plots were applied to qu­antify the inter­molecular inter­actions. The three F atoms of the tri­fluoro­methyl group are disordered over two sets of sites, with occupancy factors of 0.578 (8) and 0.422 (8). The crystal studied was refined as an inversion twin

## Abstract 2

The mol­ecule of the compound adopts the phenol–imine tautomeric form. In the crystal, mol­ecules are linked *via* pairs of bifurcated O—H⋯O hydrogen bonds between the phenol OH groups, forming inversion dimers with an 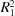
(5) ring motif.

## Chemical context   

Schiff bases (azomethines, imines) belong to a widely used group of organic compounds or inter­mediates that are important for production of certain chemical specialties, e.g. pharmaceuticals, or additives to rubber. The synthesis involves an aromatic amine and an aldehyde (Schiff *et al.*, 1881 ▸). Schiff bases are also employed as catalyst carriers (Grigoras *et al.*, 2001 ▸), thermo-stable materials (Vančo *et al.*, 2004 ▸), metal–cation complexing agents or in biological systems (Taggi *et al.*, 2002 ▸). Furthermore, they are used as starting materials in the synthesis of significant drugs with properties such as anti­fungal, anti­bacterial, anti­malarial, anti­proliferative, anti-inflammatory, anti­viral, and anti­pyretic (Hadjoudis *et al.*, 1987 ▸). On an industrial scale, they have a wide range of applications such as dyes and pigments.

In general, Schiff bases display two possible tautomeric forms, *viz*. phenol–imine and keto–amine. Depending on the tautomers, two types of intra­molecular hydrogen bonds are observed in Schiff bases: O—H⋯N in phenol–imine and N—H⋯O in keto-amine tautomers. In the present study, a new Schiff base, (*Z*)-3-({[3-meth­oxy-5-(tri­fluoro­meth­yl)phen­yl]imino}­meth­yl)benzene-1,2-diol, was obtained in crystalline form from the reaction of 2,3-di­hydroxy­benzaldehyde with 3-meth­oxy-5-(tri­fluoro­meth­yl)aniline.

## Structural commentary   

**Figure 1:**
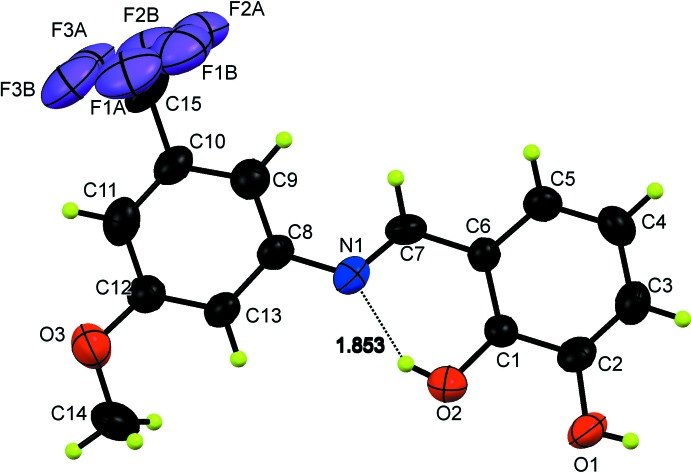
The mol­ecular structure of the title compound with the atomic numbering scheme. The dashed line shows the intra­molecular O—H⋯N hydrogen bond. Displacement ellipsoids are drawn at the 50% probability level.

The title compound crystallizes as the phenol–imine tautomer with one mol­ecule in the asymmetric unit (Fig. 1 ▸). The two phenyl rings of the Schiff base (C1–C6 and C8–C13) are inclined at an angle of 4.91 (1)° with respect to one another. The orientation of the two hy­droxy groups with respect to their tautomeric counterparts is defined by the torsion angles *T*1(C1—C6—C7—N1) and *T*2(C7—N1—C8—C9). The respective values of = 2.0 (10) and −5.5 (11)° indicate that the mol­ecule is not planar (Ünver *et al.*, 2016 ▸).
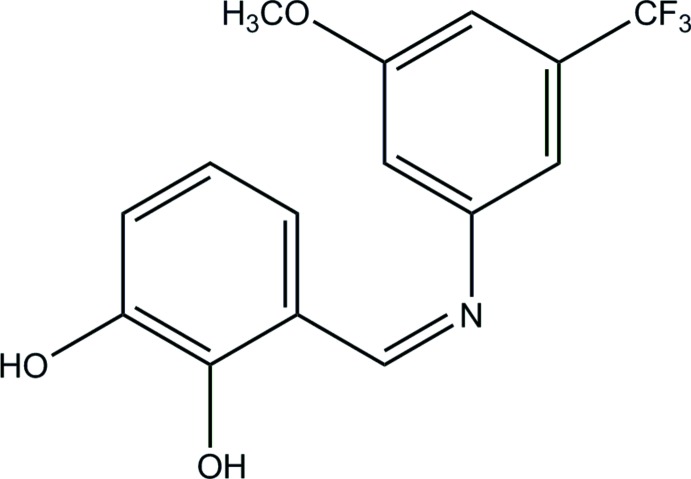


In the mol­ecule, the C=N group has a strong electron-withdrawing character as revealed by the double-bond character of N1=C7 [1.269 (8) Å] and the single bond character of O1—C2 [1.368 (6) Å] in the phenol–imine tautomer. These values and other bond lengths and angles (Table 1 ▸) are in good agreement with those previously reported for C=N and O—C bonds (Koşar *et al.*., 2010 ▸; Demir Kanmazalp *et al.*., 2019 ▸). One of the hy­droxy groups (O2) makes a strong intra­molecular O—H⋯N bond to the imine N atom (Fig. 1 ▸, Table 2 ▸) with an *S*(6) ring motif, characteristic of *o*-hy­droxy­salicyl­idene systems. Other intra­molecular hydrogen bonding inter­actions involve the disordered –CF_3_ group and adjacent aromatic H atoms bonded to C9 and C11 (Table 2 ▸). As a result of the strongly bent C—H⋯F angles of about 100°, these contributions are of minor importance.

## Supra­molecular features   

**Figure 2:**
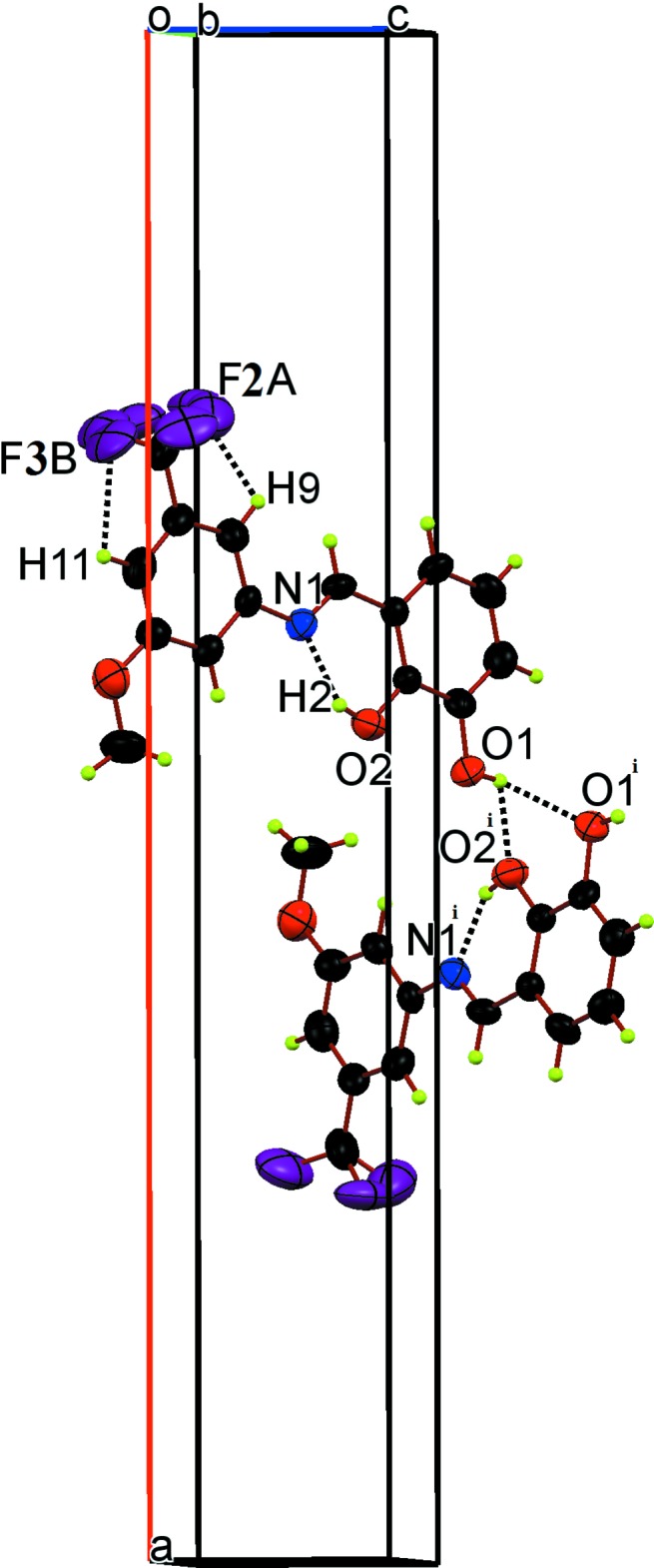
Unit-cell packing diagram for the title compound. Intra- and inter­molecular hydrogen bonds are shown as dashed lines. [Symmetry code: (i) −*x* − 1, −*y*, *z* − 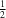.]

Between adjacent mol­ecules there are bifurcated inter­molecular O1—H⋯(O1,O2) hydrogen bonds with an 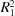
(5) graph-set motif (Fig. 2 ▸, Table 2 ▸), leading to the formation of chains parallel to [001]. Despite the presence of aromatic systems, the mol­ecule is arranged in such a way that π–π or C—H⋯π inter­actions are not favoured.

## Database survey   

A search of the Cambridge Structural Database (CSD, version 5.40, update Nov 2018; Groom *et al.*, 2016 ▸) for the (*Z*)-1-phenyl-*N*-[3-(tri­fluoro­meth­yl)phen­yl]methanimine skeleton yielded eight matches. Distinctive bond lengths (here N1=C7, C1—O2) in the Schiff base structure are the same within standard uncertainties as those of the corresponding bond lengths in the structures of 4*N*-[3,5-bis­(tri­fluoro­meth­yl)phen­yl]-3-meth­oxy­salicylaldimine (Karadayı *et al.*, 2003 ▸), 2-{[3,5-bis­(tri­fluoro­meth­yl)phen­yl]carbonoimido­yl}phenol (Yıldız *et al.*, 2015 ▸), 2-{[3,5-bis­(tri­fluoro­meth­yl)phen­yl]carbonoimido­yl}phenol (Ünver *et al.*, 2016 ▸), (*E*)-3-{[3-(tri­fluoro­meth­yl)phenyl­imino]­meth­yl}benzene-1,2-diol (Koşar *et al.*, 2010 ▸), 2-fluoro-*N*-(3-nitro­benzyl­idene)-5-(tri­fluoro­meth­yl)aniline (Yang *et al.*, 2007 ▸), (*E*)-2-methyl-6-[3-(tri­fluoro­meth­yl)-phen­yl­imino­meth­yl]phenol (Akkaya *et al.*, 2007 ▸), (*E*)-2-[(4-chloro­phen­yl)imino­meth­yl]-4-(tri­fluoro­meth­oxy)phenol (Tüf­ekçi *et al.*, 2009 ▸) and (*E*)-4-methyl-2-[3-(tri­fluoro­meth­yl)phen­yl­imino­meth­yl]phenol (Gül *et al.*, 2007 ▸). The C=N bond lengths in these structures vary from 1.270 (3) to 1.295 (5) Å and the C—O bond lengths from 1.336 (5) to 1.366 (2) Å. The mol­ecular conformations of these structures are also not planar, with dihedral angles between the phenyl rings varying between 5.00 (5) and 47.62 (9)°. It is likely that the intra­molecular O—H⋯N hydrogen bond, where the imine N atom acts as an hydrogen-bond acceptor, is an important prerequisite for the tautomeric shift toward the phenol–imine form. In fact, in all eight structures of the phenol–imine tautomers, hydrogen bonds of this type are observed.

## Hirshfeld surface analysis   

**Figure 3:**
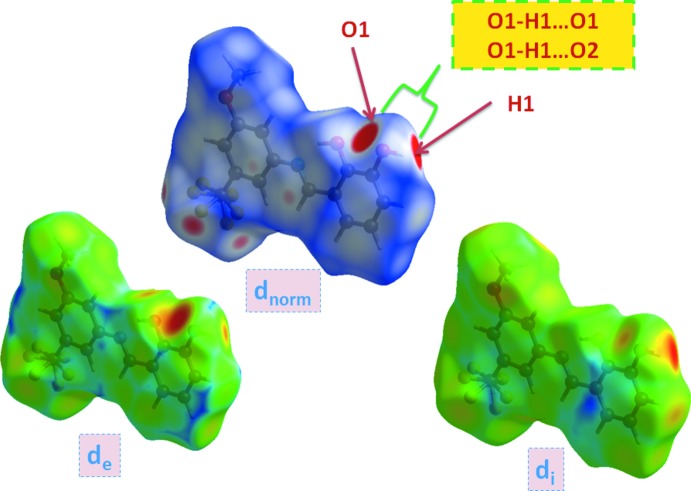
View of the three-dimensional Hirshfeld surface of the title compound plotted over *d*_norm_ (in the range −0.211 to 1.077 a.u.), *d*_e_ and *d*_i_.

**Figure 4:**
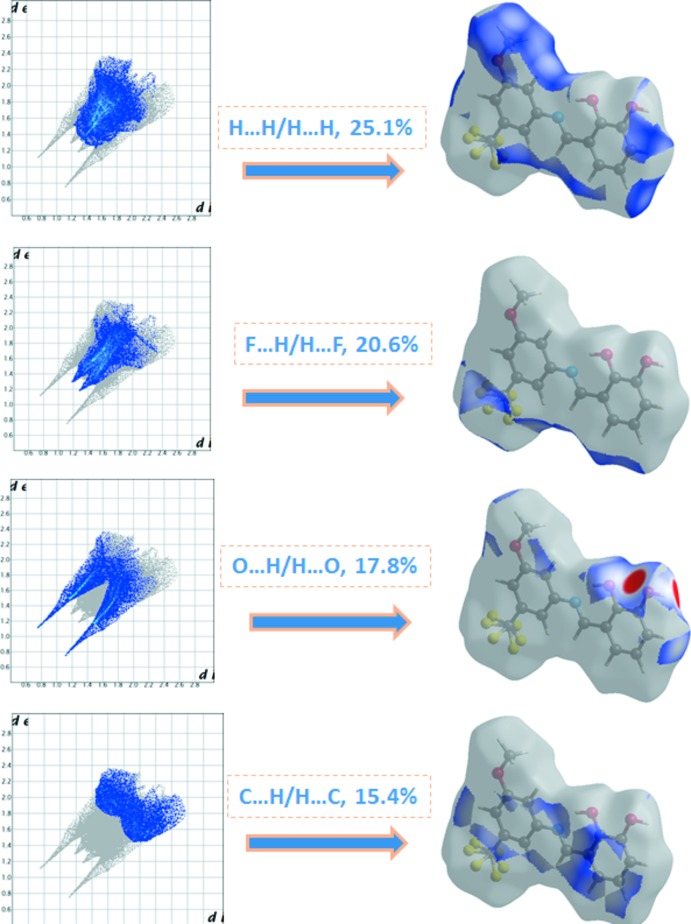
Two-dimensional fingerprint plots of the crystal with the relative contributions of the atom pairs to the Hirshfeld surface.

**Figure 5:**
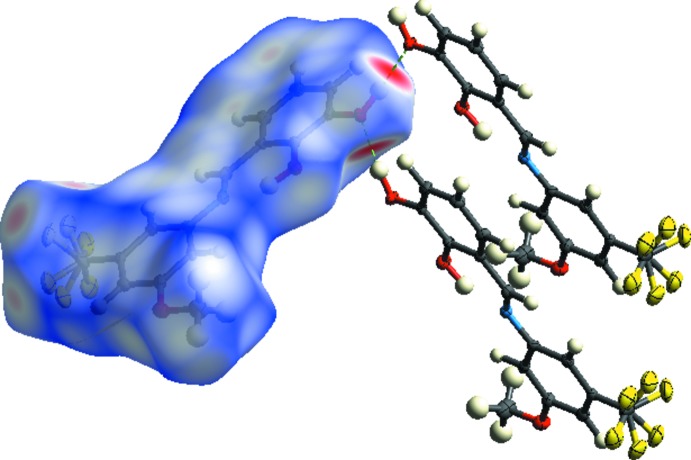
Hirshfeld surface mapped over *d*_norm_ to visualize the inter­molecular inter­actions.

Hirshfeld surface analysis of the title compound was performed utilizing the *CrystalExplorer* program (Turner *et al.*, 2017 ▸). The three-dimensional *d*_norm_ surface is a useful tool for analysing and visualizing the inter­molecular inter­actions and utilizes the function of the normalized distances *d*_e_ and *d*_i_, where *d*_e_ and *d*_i_ are the distances from a given point on the surface to the nearest atom outside and inside, respectively. The blue, white and red colour convention used for the *d*_norm_-mapped Hirshfeld surfaces indicates the inter­atomic contacts longer, equal to or shorter than the van der Waals separations. The standard-resolution mol­ecular 3D (*d*_norm_) plot with *d*_e_ and *d*_i_ for the title compound is shown in Fig. 3 ▸. The bright-red spots near the oxygen and hydrogen atoms indicate donors and acceptors of a potential O—H⋯O inter­action. As can be seen from the two-dimensional fingerprint plots (scattering points spread up to *d*_e_ = *d*_i_ = 1.5 Å; Fig. 4 ▸), the dominant inter­action in the title compound originates from H⋯H contacts, which are the major contributor (25.1%) to the total Hirshfeld surface. The contribution from the O⋯H/H⋯O contacts, corresponding to medium O1—H1⋯O1 and O1—H1⋯O2 inter­molecular inter­actions (9.6% + 8.2% = 17.8%), is represented by a pair of sharp spikes that are the characteristics of hydrogen-bonding inter­actions (Fig. 4 ▸). Other significant inter­actions are F⋯H/H⋯F (20.6%) and C⋯H/H⋯C (15.4%). While it is likely there are other identifiable points of contact that can be highlighted in the crystal, these may be of limited significance and do not require detailed discussion nor illustration. The inter­actions are visualized in Fig. 5 ▸.

## Synthesis and crystallization   

The title compound was prepared by refluxing mixed solutions of 2,3-di­hydroxy­benzaldehyde (34.5 mg, 0.25 mmol) in ethanol (15 ml) and 3-meth­oxy-5-(tri­fluoro­meth­yl)aniline (47.8 mg, 0.25 mmol) in ethanol (15 ml). The reaction mixture was stirred for 5 h under reflux. Single crystals of the title compound for X-ray analysis were obtained by slow evaporation of an ethanol solution (yield 65%, m.p. 396–398 K).

## Refinement   

Crystal data, data collection and structure refinement details are summarized in Table 3 ▸. The carbon-bound H atoms were positioned with idealized geometry and refined isotropically with C—H distances of 0.93–0.96 Å and *U*_iso_(H) set to 1.2–1.5*U*_eq_(C), and with O—H = 0.82 Å and *U*_iso_(H) = 1.5*U*_eq_(O). The three F atoms of the tri­fluoro­methyl group are disordered over two sets of sites, with occupancy factors of 0.578 (8) for F atoms with suffix *A* and 0.422 (8) for those with suffix *B* (Fig. 1 ▸). A similar behaviour for a disordered –CF_3_ group was observed in a previous study (Demir *et al.*, 2006 ▸). Restraints (SIMU, DELU and ISOR; Sheldrick *et al.*, 2015*b* ▸) were finally applied to the disordered tri­fluoro­methyl group. As a result of missing anomalous dispersion, the absolute structure of the title compound could not be determined reliably (Table 3 ▸).

## Supplementary Material

Crystal structure: contains datablock(s) I, global. DOI: 10.1107/S2056989019003220/wm5488sup1.cif

Structure factors: contains datablock(s) I. DOI: 10.1107/S2056989019003220/wm5488Isup2.hkl

Supporting information file. DOI: 10.1107/S2056989019003220/wm5488Isup3.cml

CCDC reference: 1892713

Additional supporting information:  crystallographic information; 3D view; checkCIF report

## Figures and Tables

**Table 1:** Selected geometric parameters (Å, °)

O1—C2	1.368 (6)	C1—C6	1.394 (8)
O2—C1	1.350 (7)	C6—C5	1.385 (8)
N1—C7	1.269 (8)	C6—C7	1.462 (9)
N1—C8	1.409 (8)		
			
C12—O3—C14	116.5 (6)	C9—C8—N1	125.6 (7)
C7—N1—C8	123.1 (6)	N1—C7—C6	122.1 (6)
			
C7—N1—C8—C9	−5.5 (11)	C1—C6—C7—N1	2.0 (10)
C5—C6—C7—N1	−178.7 (7)		

**Table 2:** Hydrogen-bond geometry (Å, °)

*D*—H⋯*A*	*D*—H	H⋯*A*	*D*⋯*A*	*D*—H⋯*A*
O1—H1⋯O1^i^	0.82	1.99	2.770 (4)	159
O1—H1⋯O2^i^	0.82	2.72	3.190 (6)	118
O2—H2⋯N1	0.82	1.85	2.581 (7)	147
C9—H9⋯F2*A*	0.93	2.44	2.757 (12)	100
C11—H11⋯F3*B*	0.93	2.39	2.725 (18)	101

Symmetry code: (i) 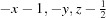.

**Table 3:** Experimental details

Crystal data	
Chemical formula	C_15_H_12_F_3_NO_3_
*M*_r_	311.26
Crystal system, space group	Orthorhombic, *P* *n* *a*2_1_
Temperature (K)	296
*a*, *b*, *c* (Å)	30.790 (3), 9.0703 (6), 4.8579 (3)
*V* (Å^3^)	1356.69 (17)
*Z*	4
Radiation type	Mo *K*α
μ (mm^−1^)	0.13
Crystal size (mm)	0.79 × 0.32 × 0.05

Data collection	
Diffractometer	Stoe IPDS 2
Absorption correction	Integration (*X-RED32*; Stoe & Cie, 2002 ▸)
*T*_min_, *T*_max_	0.957, 0.995
No. of measured, independent and observed [*I* > 2σ(*I*)] reflections	7150, 2140, 1110
*R*_int_	0.099
(sin θ/λ)_max_ (Å^−1^)	0.595

Refinement	
*R*[*F* ^2^ > 2σ(*F* ^2^)], *wR*(*F* ^2^), *S*	0.062, 0.088, 0.95
No. of reflections	2140
No. of parameters	231
No. of restraints	73
H-atom treatment	H-atom parameters constrained
Δρ_max_, Δρ_min_ (e Å^−3^)	0.14, −0.16
Absolute structure	Refined as an inversion twin
Absolute structure parameter	3 (3)

Computer programs: *X-AREA* and *X-RED32* (Stoe & Cie, 2002 ▸), *SHELXT2018* (Sheldrick, 2015*a* ▸), *SHELXL2018* (Sheldrick, 2015*b* ▸), *Mercury* (Macrae *et al.*, 2006 ▸), *WinGX* (Farrugia, 2012 ▸) and *PLATON* (Spek, 2009 ▸).

## supplementary crystallographic information

### Crystal data

**Table:**

C_15_H_12_F_3_NO_3_	*D*_x_ = 1.524 Mg m^−^^3^
*M**_r_* = 311.26	Mo *K*α radiation, λ = 0.71073 Å
Orthorhombic, *P**n**a*2_1_	Cell parameters from 5897 reflections
*a* = 30.790 (3) Å	θ = 2.2–27.0°
*b* = 9.0703 (6) Å	µ = 0.13 mm^−^^1^
*c* = 4.8579 (3) Å	*T* = 296 K
*V* = 1356.69 (17) Å^3^	Prism, red
*Z* = 4	0.79 × 0.32 × 0.05 mm
*F*(000) = 640	

### Data collection

**Table:**

Stoe IPDS 2 diffractometer	2140 independent reflections
Radiation source: sealed X-ray tube, 12 x 0.4 mm long-fine focus	1110 reflections with *I* > 2σ(*I*)
Plane graphite monochromator	*R*_int_ = 0.099
Detector resolution: 6.67 pixels mm^-1^	θ_max_ = 25.0°, θ_min_ = 2.3°
rotation method scans	*h* = −30→36
Absorption correction: integration (X-RED32; Stoe & Cie, 2002)	*k* = −10→10
*T*_min_ = 0.957, *T*_max_ = 0.995	*l* = −4→5
7150 measured reflections	

### Refinement

**Table:**

Refinement on *F*^2^	Hydrogen site location: inferred from neighbouring sites
Least-squares matrix: full	H-atom parameters constrained
*R*[*F*^2^ > 2σ(*F*^2^)] = 0.062	*w* = 1/[σ^2^(*F*_o_^2^) + (0.0144*P*)^2^] where *P* = (*F*_o_^2^ + 2*F*_c_^2^)/3
*wR*(*F*^2^) = 0.088	(Δ/σ)_max_ < 0.001
*S* = 0.95	Δρ_max_ = 0.14 e Å^−^^3^
2140 reflections	Δρ_min_ = −0.16 e Å^−^^3^
231 parameters	Absolute structure: Refined as an inversion twin.
73 restraints	Absolute structure parameter: 3 (3)

### Special details

**Table:**

Geometry. All esds (except the esd in the dihedral angle between two l.s. planes) are estimated using the full covariance matrix. The cell esds are taken into account individually in the estimation of esds in distances, angles and torsion angles; correlations between esds in cell parameters are only used when they are defined by crystal symmetry. An approximate (isotropic) treatment of cell esds is used for estimating esds involving l.s. planes.
Refinement. Refined as a 2-component inversion twin.

### Fractional atomic coordinates and isotropic or equivalent isotropic displacement parameters (Å^2^)

**Table:**

	*x*	*y*	*z*	*U*_iso_*/*U*_eq_	Occ. (<1)
F1A	−0.2587 (3)	−0.7222 (12)	−0.112 (3)	0.109 (3)	0.578 (8)
F2A	−0.2437 (3)	−0.4973 (12)	−0.058 (4)	0.110 (3)	0.578 (8)
F3A	−0.2630 (4)	−0.6345 (18)	0.281 (3)	0.117 (4)	0.578 (8)
F1B	−0.2486 (5)	−0.605 (3)	−0.165 (4)	0.115 (4)	0.422 (8)
F2B	−0.2558 (4)	−0.4953 (17)	0.212 (5)	0.111 (4)	0.422 (8)
F3B	−0.2668 (6)	−0.7256 (19)	0.191 (5)	0.112 (4)	0.422 (8)
O1	−0.48182 (12)	−0.0397 (5)	−1.1221 (12)	0.0559 (13)	
H1	−0.488061	0.002147	−1.266405	0.084*	
O2	−0.45187 (14)	−0.2219 (5)	−0.7485 (11)	0.0566 (13)	
H2	−0.439356	−0.273123	−0.634283	0.085*	
O3	−0.42289 (17)	−0.6770 (5)	0.2365 (12)	0.0675 (15)	
N1	−0.38682 (18)	−0.3355 (6)	−0.4892 (13)	0.0462 (15)	
C13	−0.4043 (2)	−0.5057 (6)	−0.1300 (19)	0.0429 (18)	
H13	−0.433339	−0.485521	−0.165359	0.051*	
C8	−0.3718 (2)	−0.4343 (7)	−0.2864 (14)	0.0422 (17)	
C2	−0.4376 (2)	−0.0449 (7)	−1.0943 (16)	0.0444 (18)	
C12	−0.3936 (2)	−0.6048 (7)	0.0740 (16)	0.050 (2)	
C1	−0.4223 (2)	−0.1434 (7)	−0.8926 (14)	0.0383 (17)	
C6	−0.37787 (19)	−0.1545 (7)	−0.8426 (13)	0.0367 (17)	
C7	−0.3617 (2)	−0.2539 (7)	−0.6293 (16)	0.0453 (18)	
H7	−0.331950	−0.257523	−0.595114	0.054*	
C10	−0.3189 (2)	−0.5665 (8)	−0.0246 (15)	0.0475 (19)	
C5	−0.3498 (2)	−0.0686 (7)	−0.9968 (15)	0.0477 (19)	
H5	−0.320085	−0.075095	−0.962902	0.057*	
C11	−0.3506 (3)	−0.6359 (7)	0.1267 (16)	0.056 (2)	
H11	−0.343162	−0.703200	0.263109	0.067*	
C9	−0.3286 (2)	−0.4645 (7)	−0.2283 (15)	0.051 (2)	
H9	−0.306471	−0.417196	−0.324352	0.061*	
C3	−0.4091 (2)	0.0371 (7)	−1.2431 (17)	0.054 (2)	
H3	−0.419422	0.101663	−1.376554	0.065*	
C4	−0.3642 (2)	0.0258 (7)	−1.1982 (16)	0.053 (2)	
H4	−0.344674	0.080776	−1.302170	0.064*	
C15	−0.2733 (3)	−0.5977 (14)	0.035 (2)	0.088 (3)	
C14	−0.4674 (2)	−0.6454 (10)	0.189 (2)	0.087 (3)	
H14A	−0.475930	−0.685003	0.013742	0.130*	
H14B	−0.471687	−0.540545	0.188861	0.130*	
H14C	−0.484666	−0.689170	0.331567	0.130*	

### Atomic displacement parameters (Å^2^)

**Table:**

	*U*^11^	*U*^22^	*U*^33^	*U*^12^	*U*^13^	*U*^23^
F1A	0.078 (5)	0.107 (7)	0.142 (8)	0.048 (5)	−0.008 (6)	−0.030 (8)
F2A	0.055 (5)	0.125 (7)	0.148 (9)	0.008 (6)	−0.018 (6)	0.015 (8)
F3A	0.080 (5)	0.155 (10)	0.116 (8)	0.043 (8)	−0.034 (6)	0.014 (8)
F1B	0.074 (6)	0.138 (9)	0.133 (9)	0.034 (8)	−0.001 (7)	0.003 (9)
F2B	0.059 (6)	0.136 (8)	0.138 (8)	0.018 (7)	−0.034 (7)	−0.017 (9)
F3B	0.087 (6)	0.118 (9)	0.132 (9)	0.042 (7)	−0.029 (8)	0.010 (9)
O1	0.041 (3)	0.075 (4)	0.051 (3)	0.014 (2)	−0.006 (3)	0.009 (3)
O2	0.043 (3)	0.067 (3)	0.060 (4)	0.002 (2)	0.003 (3)	0.012 (3)
O3	0.072 (4)	0.067 (3)	0.063 (4)	−0.011 (3)	0.002 (4)	0.012 (3)
N1	0.045 (3)	0.049 (3)	0.044 (4)	0.009 (3)	−0.004 (3)	0.000 (3)
C13	0.042 (4)	0.037 (4)	0.050 (5)	0.005 (3)	−0.009 (4)	0.000 (4)
C8	0.042 (4)	0.052 (4)	0.032 (5)	0.001 (3)	−0.006 (4)	−0.003 (4)
C2	0.039 (4)	0.047 (4)	0.047 (5)	0.013 (3)	0.004 (4)	−0.004 (4)
C12	0.046 (5)	0.049 (4)	0.057 (6)	0.002 (3)	0.000 (5)	0.002 (4)
C1	0.035 (4)	0.044 (4)	0.036 (4)	0.002 (3)	0.004 (4)	0.002 (3)
C6	0.040 (4)	0.036 (4)	0.034 (5)	−0.001 (3)	−0.002 (4)	0.000 (4)
C7	0.030 (3)	0.055 (4)	0.051 (5)	0.000 (3)	−0.004 (4)	−0.004 (4)
C10	0.048 (5)	0.053 (4)	0.042 (5)	0.016 (4)	−0.003 (4)	−0.005 (4)
C5	0.045 (5)	0.050 (4)	0.048 (5)	−0.008 (4)	−0.007 (4)	0.000 (4)
C11	0.077 (6)	0.042 (4)	0.049 (5)	0.009 (4)	−0.005 (5)	−0.002 (4)
C9	0.053 (4)	0.051 (4)	0.048 (5)	0.007 (3)	0.005 (5)	−0.001 (4)
C3	0.061 (5)	0.049 (4)	0.051 (5)	0.009 (4)	−0.002 (5)	0.007 (4)
C4	0.056 (5)	0.052 (4)	0.052 (6)	−0.011 (3)	0.008 (5)	0.006 (4)
C15	0.070 (7)	0.136 (10)	0.059 (7)	0.044 (6)	−0.004 (7)	0.029 (7)
C14	0.051 (5)	0.113 (7)	0.096 (9)	−0.017 (5)	0.008 (6)	0.026 (6)

### Geometric parameters (Å, º)

**Table:**

F1A—C15	1.410 (14)	C2—C1	1.407 (9)
F2A—C15	1.364 (14)	C12—C11	1.377 (9)
F3A—C15	1.282 (15)	C1—C6	1.394 (8)
F1B—C15	1.236 (19)	C6—C5	1.385 (8)
F2B—C15	1.375 (19)	C6—C7	1.462 (9)
F3B—C15	1.40 (2)	C7—H7	0.9300
O1—C2	1.368 (6)	C10—C11	1.376 (9)
O1—H1	0.8200	C10—C9	1.387 (8)
O2—C1	1.350 (7)	C10—C15	1.462 (10)
O2—H2	0.8200	C5—C4	1.374 (9)
O3—C12	1.366 (8)	C5—H5	0.9300
O3—C14	1.419 (8)	C11—H11	0.9300
N1—C7	1.269 (8)	C9—H9	0.9300
N1—C8	1.409 (8)	C3—C4	1.403 (9)
C13—C12	1.377 (9)	C3—H3	0.9300
C13—C8	1.412 (9)	C4—H4	0.9300
C13—H13	0.9300	C14—H14A	0.9600
C8—C9	1.389 (8)	C14—H14B	0.9600
C2—C3	1.360 (9)	C14—H14C	0.9600
			
C2—O1—H1	109.5	C10—C11—C12	119.3 (7)
C1—O2—H2	109.5	C10—C11—H11	120.3
C12—O3—C14	116.5 (6)	C12—C11—H11	120.3
C7—N1—C8	123.1 (6)	C10—C9—C8	118.9 (7)
C12—C13—C8	121.2 (6)	C10—C9—H9	120.6
C12—C13—H13	119.4	C8—C9—H9	120.6
C8—C13—H13	119.4	C2—C3—C4	120.9 (7)
C9—C8—N1	125.6 (7)	C2—C3—H3	119.5
C9—C8—C13	118.5 (7)	C4—C3—H3	119.5
N1—C8—C13	115.9 (6)	C5—C4—C3	118.4 (7)
C3—C2—O1	124.9 (7)	C5—C4—H4	120.8
C3—C2—C1	120.1 (6)	C3—C4—H4	120.8
O1—C2—C1	115.0 (6)	F3A—C15—F2A	108.6 (13)
O3—C12—C11	115.4 (6)	F1B—C15—F2B	106.8 (16)
O3—C12—C13	124.9 (6)	F1B—C15—F3B	107.0 (14)
C11—C12—C13	119.7 (7)	F2B—C15—F3B	99.5 (14)
O2—C1—C6	122.2 (6)	F3A—C15—F1A	100.6 (11)
O2—C1—C2	118.1 (6)	F2A—C15—F1A	98.8 (11)
C6—C1—C2	119.7 (6)	F1B—C15—C10	116.4 (12)
C5—C6—C1	118.6 (6)	F3A—C15—C10	118.3 (10)
C5—C6—C7	121.1 (6)	F2A—C15—C10	116.5 (9)
C1—C6—C7	120.2 (6)	F2B—C15—C10	111.7 (10)
N1—C7—C6	122.1 (6)	F3B—C15—C10	113.9 (13)
N1—C7—H7	119.0	F1A—C15—C10	111.1 (10)
C6—C7—H7	119.0	O3—C14—H14A	109.5
C11—C10—C9	122.2 (7)	O3—C14—H14B	109.5
C11—C10—C15	119.2 (8)	H14A—C14—H14B	109.5
C9—C10—C15	118.5 (8)	O3—C14—H14C	109.5
C4—C5—C6	122.2 (7)	H14A—C14—H14C	109.5
C4—C5—H5	118.9	H14B—C14—H14C	109.5
C6—C5—H5	118.9		
			
C7—N1—C8—C9	−5.5 (11)	O3—C12—C11—C10	−179.1 (6)
C7—N1—C8—C13	173.7 (7)	C13—C12—C11—C10	0.5 (11)
C12—C13—C8—C9	−0.8 (11)	C11—C10—C9—C8	−1.7 (11)
C12—C13—C8—N1	179.9 (6)	C15—C10—C9—C8	180.0 (8)
C14—O3—C12—C11	179.2 (7)	N1—C8—C9—C10	−179.0 (6)
C14—O3—C12—C13	−0.3 (11)	C13—C8—C9—C10	1.7 (11)
C8—C13—C12—O3	179.1 (7)	O1—C2—C3—C4	179.2 (7)
C8—C13—C12—C11	−0.3 (11)	C1—C2—C3—C4	−0.2 (12)
C3—C2—C1—O2	179.6 (7)	C6—C5—C4—C3	1.5 (11)
O1—C2—C1—O2	0.0 (9)	C2—C3—C4—C5	−1.0 (12)
C3—C2—C1—C6	1.1 (11)	C11—C10—C15—F1B	141.8 (17)
O1—C2—C1—C6	−178.4 (6)	C9—C10—C15—F1B	−40 (2)
O2—C1—C6—C5	−179.1 (6)	C11—C10—C15—F3A	−28.9 (18)
C2—C1—C6—C5	−0.7 (10)	C9—C10—C15—F3A	149.5 (13)
O2—C1—C6—C7	0.3 (10)	C11—C10—C15—F2A	−161.1 (11)
C2—C1—C6—C7	178.6 (6)	C9—C10—C15—F2A	17.3 (17)
C8—N1—C7—C6	178.7 (6)	C11—C10—C15—F2B	−95.2 (15)
C5—C6—C7—N1	−178.7 (7)	C9—C10—C15—F2B	83.2 (14)
C1—C6—C7—N1	2.0 (10)	C11—C10—C15—F3B	16.6 (18)
C1—C6—C5—C4	−0.6 (11)	C9—C10—C15—F3B	−165.0 (13)
C7—C6—C5—C4	−179.9 (6)	C11—C10—C15—F1A	86.8 (12)
C9—C10—C11—C12	0.6 (11)	C9—C10—C15—F1A	−94.9 (13)
C15—C10—C11—C12	178.9 (8)		

### Hydrogen-bond geometry (Å, º)

**Table:**

*D*—H···*A*	*D*—H	H···*A*	*D*···*A*	*D*—H···*A*
O1—H1···O1^i^	0.82	1.99	2.770 (4)	159
O1—H1···O2^i^	0.82	2.72	3.190 (6)	118
O2—H2···N1	0.82	1.85	2.581 (7)	147
C9—H9···F2*A*	0.93	2.44	2.757 (12)	100
C11—H11···F3*B*	0.93	2.39	2.725 (18)	101

Symmetry code: (i) −*x*−1, −*y*, *z*−1/2.
